# Weed or Wheel! fMRI, Behavioural, and Toxicological Investigations of How Cannabis Smoking Affects Skills Necessary for Driving

**DOI:** 10.1371/journal.pone.0052545

**Published:** 2013-01-02

**Authors:** Giovanni Battistella, Eleonora Fornari, Aurélien Thomas, Jean-Frédéric Mall, Haithem Chtioui, Monique Appenzeller, Jean-Marie Annoni, Bernard Favrat, Philippe Maeder, Christian Giroud

**Affiliations:** 1 Department of Radiology, Centre Hospitalier Universitaire Vaudois (CHUV), and University of Lausanne, Lausanne, Switzerland; 2 CIBM (Centre d’Imagerie Biomédicale), Centre Hospitalier Universitaire Vaudois (CHUV) unit, Lausanne, Switzerland; 3 CURML (University Center of Legal Medicine), UTCF (Forensic Toxicology and Chemistry Unit), Geneva, Switzerland; 4 Department of Psychiatry, SUPAA (Service Universitaire de Psychiatrie de l’Age Avancé), Centre Hospitalier Universitaire Vaudois (CHUV), Lausanne, Switzerland; 5 Department of Clinical Pharmacology and Toxicology, Centre Hospitalier Universitaire Vaudois (CHUV), Lausanne, Switzerland; 6 Neurology Unit, Department of Medicine, University of Fribourg, Fribourg, Switzerland; 7 CURML (University Center of Legal Medicine), UMPT (Unit of Psychology and Traffic Medicine), Lausanne and Geneva, Switzerland; 8 CURML (University Center of Legal Medicine), UTCF (Forensic Toxicology and Chemistry Unit), Lausanne, Switzerland; Peking University, China

## Abstract

Marijuana is the most widely used illicit drug, however its effects on cognitive functions underling safe driving remain mostly unexplored. Our goal was to evaluate the impact of cannabis on the driving ability of occasional smokers, by investigating changes in the brain network involved in a tracking task. The subject characteristics, the percentage of Δ^9^-Tetrahydrocannabinol in the joint, and the inhaled dose were in accordance with real-life conditions. Thirty-one male volunteers were enrolled in this study that includes clinical and toxicological aspects together with functional magnetic resonance imaging of the brain and measurements of psychomotor skills. The fMRI paradigm was based on a visuo-motor tracking task, alternating active tracking blocks with passive tracking viewing and rest condition. We show that cannabis smoking, even at low Δ^9^-Tetrahydrocannabinol blood concentrations, decreases psychomotor skills and alters the activity of the brain networks involved in cognition. The relative decrease of Blood Oxygen Level Dependent response (BOLD) after cannabis smoking in the anterior insula, dorsomedial thalamus, and striatum compared to placebo smoking suggests an alteration of the network involved in saliency detection. In addition, the decrease of BOLD response in the right superior parietal cortex and in the dorsolateral prefrontal cortex indicates the involvement of the Control Executive network known to operate once the saliencies are identified. Furthermore, cannabis increases activity in the rostral anterior cingulate cortex and ventromedial prefrontal cortices, suggesting an increase in self-oriented mental activity. Subjects are more attracted by intrapersonal stimuli (“self”) and fail to attend to task performance, leading to an insufficient allocation of task-oriented resources and to sub-optimal performance. These effects correlate with the subjective feeling of confusion rather than with the blood level of Δ^9^-Tetrahydrocannabinol. These findings bolster the zero-tolerance policy adopted in several countries that prohibits the presence of any amount of drugs in blood while driving.

## Introduction

Drug use and drug-alcohol combinations increase the risk of traffic accidents [1], [2], [3]. However, decrease of perceptual motor control [4], motor inhibition and cognition [5] under cannabis intoxication were subtle [6], [7] or more prominent [1] and the correlated risk in driving does not reach a consensus [3]. These discrepancies can be partially explained by differences in dosage, experimental setting, and demographic characteristics of the tested subjects [8], [9]. Furthermore, in order to succeed in a task under the effects of cannabis, a subject can either increase brain activation of the same network or rely on different supplementary networks – i.e. integrating different strategies. Demonstration of networks modification after Δ^9^-Tetrahydrocannabinol (THC) inhalation requires an additional sophisticated imaging approach, such as PET investigation or functional magnetic resonance imaging of the brain (fMRI) [10]. It has been shown that the impairing effects of cannabis may happen even with very low blood levels of THC and that complex concentration-effects relationships and pharmacokinetics might preclude using a particular THC blood threshold to make fair legal determinations of impairment [11].

Taking this into consideration, different prevention/deterrence strategic initiatives have been adopted to reduce traffic accidents related to cannabis abuse. Switzerland (in 2005) and several other European countries have adopted the “zero tolerance policy” that prohibits the presence of any amount of drugs in the blood while driving [12]. A Swiss nationwide study (2005) carried out on whole blood samples from drivers suspected of driving under the influence of drugs pointed to the prevalence and severity of this problem by revealing that one or more psychoactive drugs were detected in 89% of all analyzed samples [13]. The same study showed that the most frequently encountered drugs were cannabinoids (for 48% of the total number of cases and for a large majority of young male drivers).

The necessity to regulate the consumption of cannabis while driving is obvious. In this context the European Union established DRUID (Driving under the Influence of Drugs, Alcohol and Medicines), a European project with the objective of giving scientific support to the transport policy to combat impaired drivers. The mechanism by which the use of cannabis causes a decrease in the ability to drive is still poorly understood. Recent brain imaging studies mostly focus on the assessment of long term consequences of cannabis use and have led to conflicting results [14]. Some fMRI investigations showed hyperactivation [15], [16] while others showed hypoactivation [17], [18] in prefrontal, frontal, and cerebellar brain areas. An explanation of the discrepancy of the results could be attributed to test and population characteristics (cognitive demands required by the task and abstinent/just intoxicated cannabis users), inter-individual differences linked to personality (temperament, level of anxiety or arousal), or drug-related factors such as recency of use or the quantity of drug used on an everyday basis [14], [19]. Despite this, some common features emerge.

Several studies have shown that cannabis increases global brain perfusion (CBF) [20], [21], and this must be considered in the design of functional brain studies. PET studies on the immediate effects of cannabis revealed regional differences in rCBF in the frontal, insular, and anterior cingulate cortices, as well as in the cerebellum [22]. However, the investigations of the function of the brain of just-intoxicated cannabis users remain scarce compared to the literature devoted to addicted ones [23].

The aim of this study was to investigate the acute effects of smoking high-potency cannabis joints on psychomotor skills related to driving. To this end, we analysed the mutual effects and interactions among blood levels of cannabinoids, changes in brain network activations, psychomotor skills, and clinical and subjective effects. A standardized experimental setting was of paramount importance and included a placebo-controlled cross-over design and a fixed-pace inhalation procedure. We assessed subjects’ cognitive control abilities crucial for safe driving through an fMRI experiment during a pursuit tracking task. We also determined whole blood cannabinoids time profiles.

The brain networks involved in a tracking task are documented [24], [25] and encompass several areas that support cognitive control for selecting, switching, and attending to salient events in the environment. We hypothesize that cannabis alters the normal activity of these circuits and the aim of the study was to map these modulations due to drug exposure and assess if these changes are global or local. We consider the forensic implications of the observed modifications in brain activations and task performances.

## Methods

### 2.1. Ethics Statement

The study was approved by the Cantonal Research Ethics Committee (Vaud). The subjects gave written informed consent and received financial compensation for their participation.

### 2.2. Subjects and Recruitment

Thirty-one healthy male volunteers between eighteen and thirty years of age, all occasional cannabis smokers, participated in the study. Subjects were recruited through advertisements in hospitals and universities. All participants were right-handed with normal or corrected-to-normal vision, and had no known history of neurological or psychiatric disorders. Handedness was assessed using the Edinburgh Handedness Inventory and by visual check while subjects were performing the tasks. For the Critical Tracking Task (CTT), they all spontaneously used the right hand for holding the joystick.

The inclusion protocol consisted of several distinct steps: during a first interview, we gave detailed explanations about the experimental protocol and we encouraged the subjects to discuss all the potential positive and negative side-effects of cannabis smoking. Subjects then underwent a thorough medical examination and a psychiatric interview.

The psychiatric interview was based on the AMDP system (Arbeitsgemeinschaft für Methodik und Dokumentation in der Psychiatrie) [26] with the DSM-IV-TR diagnostic criteria (Diagnostic and statistical manual of mental disorders, fourth edition, text revision). Subjects with a current (or a history of) psychiatric disorder on axes I or II were excluded. They were also evaluated with the Global Assessment of Functioning scale (GAF) (DSM-IV-TR), the Montreal Cognitive Assessment (MoCA) screening tool for mild cognitive impairment [27] (score below 26/30 was the exclusion criterion), and a modified Cannabis Abuse Screening test (CAST) [28].

During this inclusion visit, participants provided a detailed medical history and filled out a questionnaire about their drug use and habits. The mean consumption of cannabis for the 3 months preceding inclusion in the study was set to a minimum of one joint per month and a maximum of less than one joint per week. We presented the volunteers with a diagram showing various mixtures of tobacco and cannabis in a cigarette in order to estimate the amounts of cannabis and tobacco they usually mixed in self-made joints.

We carried out a breath alcohol test and a Syva RapidTest d.a.u.® 4 immunoassay from Dade Behring for qualitative detection of opiates, cannabis, cocaine, and amphetamines in urine as well as an ECG and a spirometry test.

A blood sample was also taken to determine the cannabinoids concentration and evaluate the volunteers’ rate of smoking (in case of positive urinary test for cannabis). Concentrations of (-)-11-nor-Δ^9^-THC-carboxylic acid (THCCOOH), the main inactive THC metabolite in whole blood, higher than 35 ng/ml were presumptive of regular cannabis smoking (more than 200 occasions per year) and were a reason for exclusion. Whole blood levels of cannabinoids were measured by means of fast gas chromatography and negative-ion chemical ionization tandem mass spectrometry. The limits of detection (LOD) ranged between 0.1 and 0.2 ng/mL for all cannabinoids. The limits of quantification were of 0.5 ng/ml for both THC and 11-OH-THC and 2.5 ng/ml for THCCOOH [29].

The volunteers who used any illegal drug other than cannabis, had abnormal clinical parameters, or presented a psychiatric history were excluded from the study.

### 2.3. Experimental Protocol

Subjects included in the study were requested to abstain from smoking cannabis and tobacco for at least twelve hours prior to the experimental days. To exclude exposure to other common drugs (opiates, cocaine and amphetamines) or alcohol, we performed a breath alcohol test and a urine immunoassay (Syva RapidTest d.a.u.® 4) at the beginning of each experimental day.

**Figure 1:**
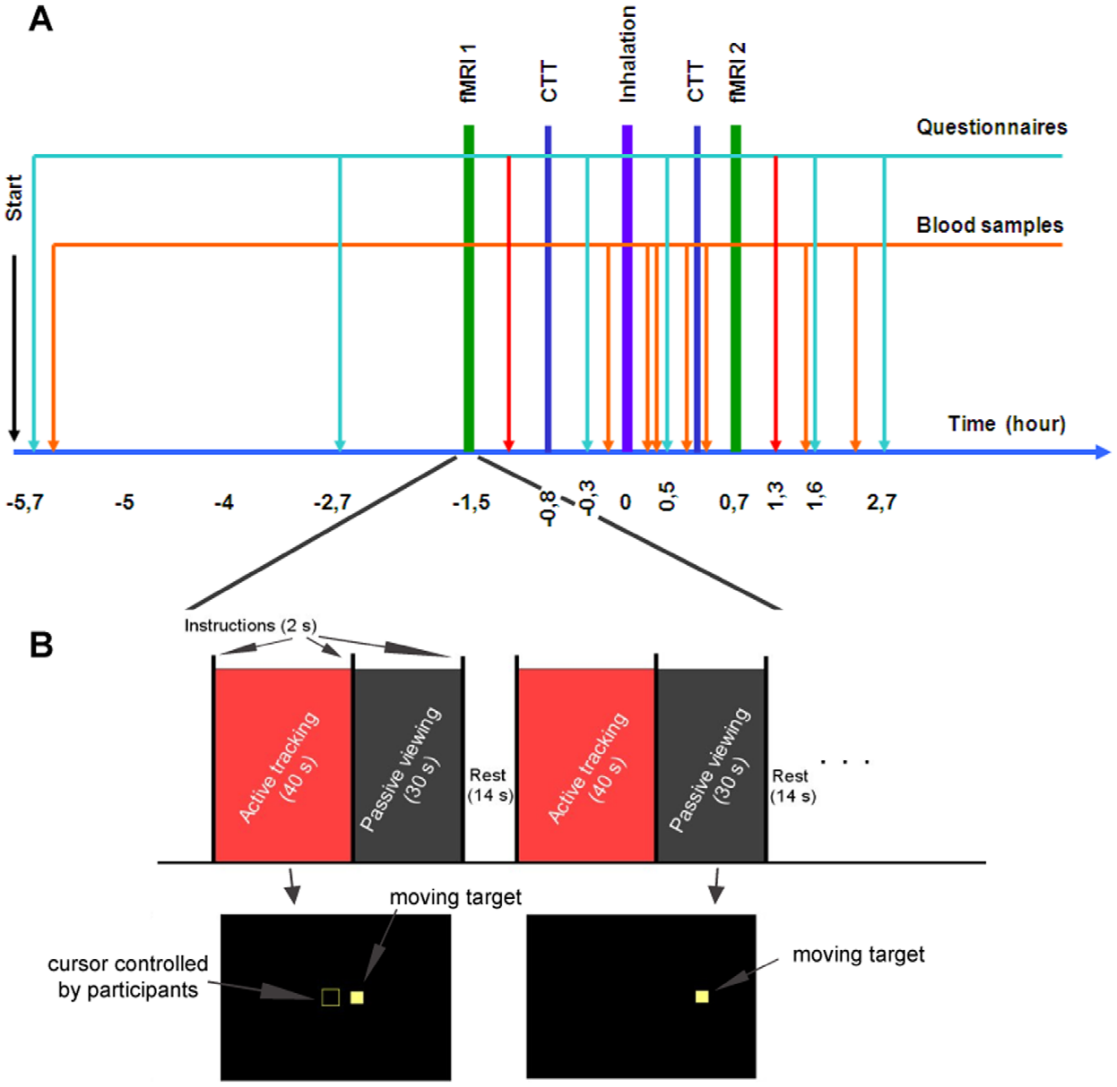
Schematic representation of the experimental day and of the fMRI task. Panel A shows the general scheme of the whole protocol. It includes eight whole blood samplings (orange arrows), 4 of them rapidly after the fixed-pace inhalation procedure (violet bar) in order to construct the kinetics of the main metabolites. Volunteers were asked to perform two fMRI experiments (green bars), once before and once after smoking the joint, and two Critical Tracking Task (CTT) outside the scanner for the assessment of psychomotor skills (blue bars). On six occasions the volunteers filled out questionnaires on the subjectively experienced effects of smoking a joint and their willingness to drive (light blue arrows). After each fMRI, the volunteers filled out another questionnaire in order to detect any change in their tactical skills and in the way they performed the tracking tests (yellow arrows). Panel B summarizes the fMRI protocol organized in a block-design fashion where each cycle of the three experimental conditions (active, passive, and rest) was repeated five times. The rest period was 14 s long, whereas active and passive conditions lasted 40 s and 30 s, respectively. At the beginning of each experimental condition, subjects received a brief visual cue (2 s) regarding the type of task they were required to perform. In the active phase subjects were asked to track the position of a target square that moved along the horizontal meridian by keeping it at the center of a user-controlled square by means of an MRI-compatible joystick (left-most illustration in the bottom part of the panel). In the passive phase subjects were instructed only to visually follow the target square movement (right-most illustration in the bottom part of the panel).

Volunteers participated in two independent cross-over experimental sessions where they smoked either a joint of pure cannabis or a placebo. The study was double-blind and the sessions were randomized, counterbalanced, and spaced one week apart. The time-schedule of each experimental day consisted of one smoking session, two fMRI tracking tasks, and two critical tracking task (CTT) tests performed on either side of the smoking time-window. On six occasions during each experiment day, the volunteers filled out questionnaires on the subjectively experienced effects of smoking a joint and their willingness to drive under various fictitious scenarios. We recorded blood pressure and heart rate and estimated eye reddening with a visual discontinuous unitary scale graded from 0 (no reddening) to 4 (bloodshot eyes). Throughout the day, blood samples were collected. The time schedule is summarized in figure 1.

Details of cannabis material (Bedrobinol, 11% THC, <1% CBD), preparation of the joint (0.7g pure cannabis), inhalation procedure (fixed paced puffing procedure, about 42 mg THC inhaled) and toxicological analyses can be found in Doc S1.

### 2.4. Questionnaires

The day of the experiment we used a set of visual analogue scales (ranging from 0 to 100) on six different occasions to assess subjective measurements of mood, drug effects, and of the willingness to drive [30]. In addition, after each fMRI session, volunteers filled a quantitative self report in order to detect any change in their tactical skills and in the way they performed the tracking tests (details in Doc S1). After controlling for the normality of the distribution of the scores, we assessed subjects’ answers under the different experimental conditions using either a paired t test or a Wilcoxon signed-ranks non-parametric test. The analysis was performed by subtracting the scores of the post smoking-THC/placebo sessions from the scores obtained after the corresponding control sessions.

### 2.5. Assessment of Psychomotor Skills

Psychomotor skills were evaluated through a Critical Tracking Task (CTT, Systems technology Inc.). The CTT is a simple, widely used, and fully validated test of psychomotor functioning, measuring eye-hand coordination and delays in visual motor response [31], [32]. It was run on a laptop equipped with a joystick, allowing the lateral control of a schematic representation of a vehicle. Participants were required to keep their car in the middle of the roadway despite an increasingly unstable first-order pseudo-erratic movement of the vehicle laterally with respect to the middle of the roadway. Each session was preceded by a training period. The CTT score λ_c_ in rad/s at the critical point of loss of control was recorded for five trials and averaged after exclusion of the lowest and the highest values [30]. The CTT is known to detect any impairment caused by fatigue, illness, alcohol, medications, or psychotropic drugs. This test had been widely used to detect psychomotor impairment after cannabis smoking [33]. Furthermore, the CTT is one of the few psychomotor tasks measuring “skills related to driving” that actually has been shown to possess a moderate correlation to real-life driving as measured in on-the-road tasks [34].

### 2.6. fMRI Tracking Task

A computerized visuo-motor pursuit tracking test (BigBeCurseur 2.2.0 from Ariacom SARL, Plan-Les-Ouates, Geneva, Switzerland) was adapted to fit an fMRI setting. Stimuli appeared in the center of a computer screen. An LCD projector equipped with a photographic zoom lens and with a refresh rate of 75 Hz displayed the stimuli on a translucent screen positioned in the back part of the bore. Subjects viewed stimuli through a custom-made mirror positioned inside the magnet and had a field of view of ±20° horizontally and ±11° vertically.

The test included three experimental conditions: active, passive, and rest (Fig. 1b). Each of the three conditions was repeated in sequence 5 times. In the active and in the passive condition a target square (size 2.8° of the field of view) moved along the horizontal meridian (excursion ±16°) following pseudo-random trajectories (linear combination of six sinus functions). In the active condition, subjects were asked to track the target position by keeping it at the center of a user-controlled square (size 3.6° of the field of view) by means of an MR compatible joystick (MAG Design & Engineering, Redwood City, CA), which they all (voluntarily) held in their right hand. The passive condition was similar to the active one, with the difference that subjects were instructed only to visually follow the target square movement. During the rest phase, the target square was positioned in the center of the screen and subjects were asked to fixate on it. Continuous behavioural variables were recorded during the active phase and are detailed in the section 2.10.

Prior to the scanning session subjects participated in a training session. They were asked to perform a few cycles of a task similar to the one that would be performed during the fMRI exam, so as to familiarize them with the procedure and to strongly diminish any learning effect during the performance session. During the training session the trajectories of the target followed a linear combination of six sinus functions having same period and amplitude of those used during the fMRI acquisition sessions. The combination of the functions in time was different in order to create a different scenario of equal difficulty level.

### 2.7. fMRI Acquisition Protocol

The fMRI acquisition was performed in a single run of 7 minutes and 30 seconds, corresponding to the acquisition of 225 volumes. The test was organized in a block-design fashion and the three experimental conditions (active, passive, and rest, Fig. 1b) constituting the cycle were repeated in this order 5 times. The baseline is represented by the rest condition. The rest period was 14 s long, whereas active and passive conditions lasted 40 s and 30 s, respectively. At the beginning of each experimental condition, subjects received a brief visual cue (2 s) regarding the type of task they were required to perform. A similar paradigm involving active and passive/watching blocks was used to assess neural systems in simulated driving [35] and their modification after alcohol intoxication [36].

FMRI was performed once before and once after each participant smoked a placebo or a cannabis joint, in order to have for each participant his own control session. The time period selected for fMRI was just after the rapid distribution phase of THC (starting about 45 min after the end of the inhalation), when cannabinoids concentrations change more slowly. This phase was also supposed to correspond to the time-period when drugged drivers are generally apprehended by police. Mean cannabinoids levels during the fMRI session were interpolated from values determined on both sides of the session.

Scanning was performed on a 3T Siemens Trio scanner equipped with a 32-channel head-coil. Imaging parameters were as follows: 1 single run, 225 images, single-shot EPI gradient echo sequence, repetition time 2000 ms, echo time 30 ms, flip angle 90°, pixel size 3×3 mm, 32 slices of 3 mm covering the whole brain (acquired in an ascending order). We prevented head movements by cushioning the participant’s head in the coil with padding.

High-resolution T1-weighted 3D gradient-echo sequence (MPRAGE), 160 slices (1×1×1 mm voxel size), was acquired as structural basis for brain segmentation and surface reconstruction.

### 2.8. Hemodynamic Response Assessment

To ensure that cannabis smoking did not affect the shape of the Hemodynamic Response Function (HRF), we extracted filtered time series from four regions of interest (3 mm radius spheres covering the primary visual cortex, the motor cortex, the insula, and the Anterior Cingulate Cortex) and used the Inverse Logit Model [37], [38], [39] to estimate the HRF as a function of the four experimental sessions (two control sessions before smoking, one session after placebo smoking, and one after cannabis smoking) for each subject. We did not observe significant differences in time-to-peak and width of the estimated response in relation to the experimental sessions. This supports the use of the standard Hemodynamic Response Function in our analysis.

### 2.9. Analysis of fMRI Data

FMRI data were pre-processed and analyzed using Statistical Parametric Mapping (SPM8, Wellcome Department of Cognitive Neurology, London, UK). Intra-session acquisitions were re-aligned to the first scan using a six-parameter rigid-body transformation. Functional images were then co-registered to the respective anatomical acquisition and normalized to the Montreal Neurological Institute template (MNI) using a 12-parameter affine transformation and a resampled voxel size of 2 mm isotropic. Images were subsequently spatially smoothed with an isotropic Gaussian kernel (FWHM = 6 mm) to increase the signal-to-noise ratio.

Single participant analysis was performed using the General Linear Model according to our specific block design experiment. The signal drift across acquisitions was removed with a high-pass filter. Statistical parametric maps of the contrasts of interest were computed for each subject modelling the active and the passive blocks and the 4 experimental conditions in the same design. Realignment parameters were included in the model as regressors. Maps were used as input values for the group statistics based on Random Field Theory. In particular the inferential statistics included a 2×2 Repeated Measures ANOVA with 2 levels per factor (Factor 1: before/after smoking levels, factor 2: Placebo/THC joint levels), and post-hoc t-tests. We considered significant only clusters surviving at p<0.05 (Family Wise Error (FWE) corrected) and for cluster extent of k>40 (greater than the minimum number of voxels expected per cluster).

Regression analyses were performed on a voxel-by-voxel basis.

### 2.10. Analysis of Psychomotor Data

Subject performance was quantified by measuring the precision of the behavioural variables, which were continuously recorded during the active phase of the experiment. Parameters of interest were the duration of correct tracking (defined as the time during which the cursor and the target were superposed for at least 50% of their surfaces) and the mean gap between target and cursor (measured in % of screen resolution). Results were averaged across the five active epochs. In order to compensate for any potential confounding effects related to training, environment, and technical equipment, we assessed behavioural changes by subtracting the performance during the post smoking-THC/placebo sessions from the performance during the corresponding control sessions (before smoking).

For each variable of interest, we tested the null hypothesis of whether the group performance followed a normal distribution using the Shapiro-Wilk test. If the supposition of normality could be accepted, changes in group performance were tested by means of Paired T-tests across sessions. Otherwise, if the null hypothesis was rejected, we performed the non-parametric Wilcoxon signed-ranks test.

## Results

Twenty-three volunteers were included in the analysis (24±3 years). A summary of their sociodemographic characteristics, history, and patterns of drug use is presented in table 1 (excluded subjects are described in Doc S1).

**Table 1:** Sociodemographic characteristics, self-rated patterns of cannabis use and subjective feeling of unwanted side-effects.

	Number	Mean	Std	Median	Maximum	Minimum	
Age		24.1	3	25	29	19	
Ethnicity	*Caucasian (21), Asian (1), Eurafrican (1)*						
Education (post-compulsory)		6	2.3	6	10	2	
Employed or Student (E)/Jobless (J)	*21 E, 2 J*						
Regular dwelling (D)/Homeless (H)	*23 D*						
Driving license	*20 (19 car, 1 motorbike)*						
Regular sport practice (Yes/No)	19 Y, 3 N						
Sociability (0–1–2)		1.3	0.7	1	2	0	
Feel healthy (0–1–2)		2	0	2	2	2	
Novel experiment seeker (Yes/No)	13 Y, 8 N						
X-Sport practitioner (Yes/No)	6 Y, 17 N						
Trait anxiety index (0–1–2–3)		0.5	0.8	0	2	0	
Number of standard alcohol drinks per week		5	2.8	5	10	1	
Age at first cannabis use		16.4	3	17	23	9	
Total years of lifetime cannabis use		7.7	3.3	7	15	4	
Preferred forms of cannabis	*Marijuana(20), Haschich (10), Haschich oil (5), Pollen (2)*						
Preferred methods of consumption	*Joint (23), Water pipe (bong, bhang) (7), Pipe (chillum, sebsi) (10), Cigar (Blunt) (3), Vaporizer (1)*						
Assessment of the usual size of a joint (grams)		0.4	0.3	0.4	1	0.1	
Estimation of the [%] of cannabis in the cannabis/tobacco mix		48	18	50	70	30	
Frequency of use (times/month, 3 last months)		3.7	2.3	3.5	10	1	
Number of people with whom the joint is shared		3.3	0.9	3.5	5	2	
Prefer light (L) or strong (S) cannabis	*13 L, 9 S*						
Usually inhale deeply (Yes/No)	*7 Y, 18 N*						
Feelings reported after smoking cannabis							
Anxiety	*rarely (7)*						
Confusion	*often (2), rarely (7)*						
Drowsiness	*often (4), rarely (13)*						
Palpitations/tachycardia	*often (3), rarely (7)*						

### 3.1. Cannabinoids Elimination Time Profiles

**Figure 2:**
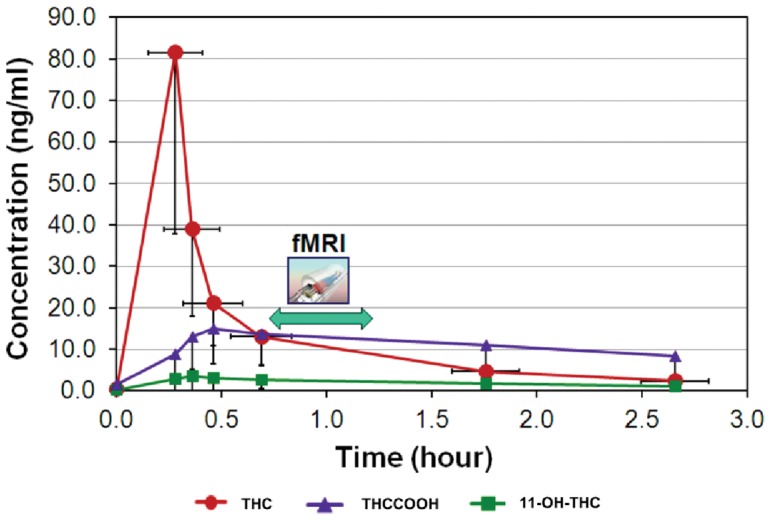
Time profiles of the major cannabinoids taken from whole blood. Time 0 corresponds to the last blood sample collected right before the smoking procedure; concentrations are expressed in ng/ml. We performed the fMRI when THC blood concentration roughly drops to one sixth of its maximum value (45 minutes after smoking). Vertical error bars represent standard deviation of the measurements, horizontal bars represent time variability in the collection of samples.

The mean kinetic profiles of the 3 main cannabinoids measured in whole blood are displayed in figure 2. The highest THC concentrations were measured in the blood sampled just after smoking. They varied considerably among subjects, with a median value of 87.4 ng/ml (range: 16.8–167.9). The whole-blood THC levels during the fMRI investigation time-period ranged from 2.9 to 23.7 ng/ml (median value: 9.3 ng/ml). This value was estimated from the THC levels measured on either side of the fMRI exam. Table 2 lists the individual highest THC, 11-OH-THC, and THCCOOH levels and their corresponding interpolated concentrations during fMRI. Compared to THC, peak concentrations for both metabolites were time-delayed (0.5 hour for THCCOOH and 0.3 hour for 11-OH-THC) and achieved much lower ranges (median value: 14.7 ng/ml for THCCOOH). The active metabolite 11-OH-THC exhibited very low peak concentrations (median value: 2.6 ng/ml, range: 1.1–17.9 ng/ml).

**Table 2:** Cannabinoids concentrations (ng/ml). Time point zero is the beginning of the inhalation procedure.

Highest concentrations	THC	11-OH-THC	THCCOOH
Number	23	23	23
Median (ng/ml)	87.4	2.6	14.7
Mean (ng/ml)	81.6	3.5	15.2
Standard deviation (ng/ml)	43.7	3.4	7.9
Highest value (ng/ml)	167.9	17.9	38.3
Lowest value (ng/ml)	16.8	1.1	4.7
Time after starting smokingthe joint (hour)	0.3	0.5	0.5
**Interpolated concentrations**	**THC**	**11-OH-THC**	**THCCOOH**
Number	23	23	23
Median (ng/ml)	9.3	1.9	11.3
Mean (ng/ml)	9.4	2.3	12.6
Standard deviation (ng/ml)	5	1.8	7.2
Highest value (ng/ml)	23.7	9.2	32.6
Lowest value (ng/ml)	2.9	0.4	3.1
Time after starting smokingthe joint (hour)	1.1	1.1	1.1

### 3.2. Self-estimation of Drug Effects with VAS

**Figure 3:**
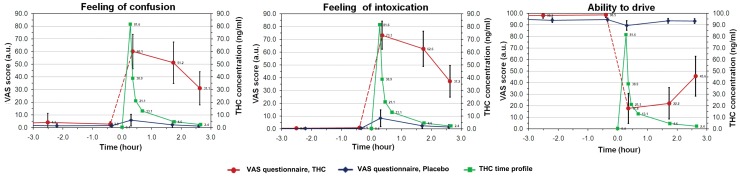
Self-evaluation of drug effects. Joint scheme of the subjective estimation of drug effects after cannabis (red curve) and placebo (blue curve) smoking evaluated by questionnaires answered using a Visual Analog Scale ranging from 0 to 100. The time profile of THC concentrations measured in whole blood (green curve, concentrations on right vertical axe) is given as reference. Subjects ratings and concentrations are averaged across subjects. Error bars represent standard deviation.

Subjects experienced positive and negative feelings for the three hours following smoking. They reported feelings of intoxication, of confusion (both shown in figure 3), of a “high,” or of the environment having changed (the last two not shown in fig. 3). When comparing the elimination time-profile of THC with these reported sensations, the alteration of these feelings persisted well beyond the peak of concentration, around three hours after smoking the joint. During this late phase, while the subjective effects were still intense, THC levels had dropped to relatively low concentrations (less than 5 ng/ml). After smoking the placebo, volunteers reported only a very slight perception of intoxication, of confusion, and of environmental changes. The ability to drive showed a similar trend (fig. 3). A considerable and long-lasting decrease in the subjective feeling of being able to drive was reported after smoking the joint, while only a tiny decrease was observed after smoking the placebo.

### 3.3. Strategy Questionnaires

**Figure 4:**
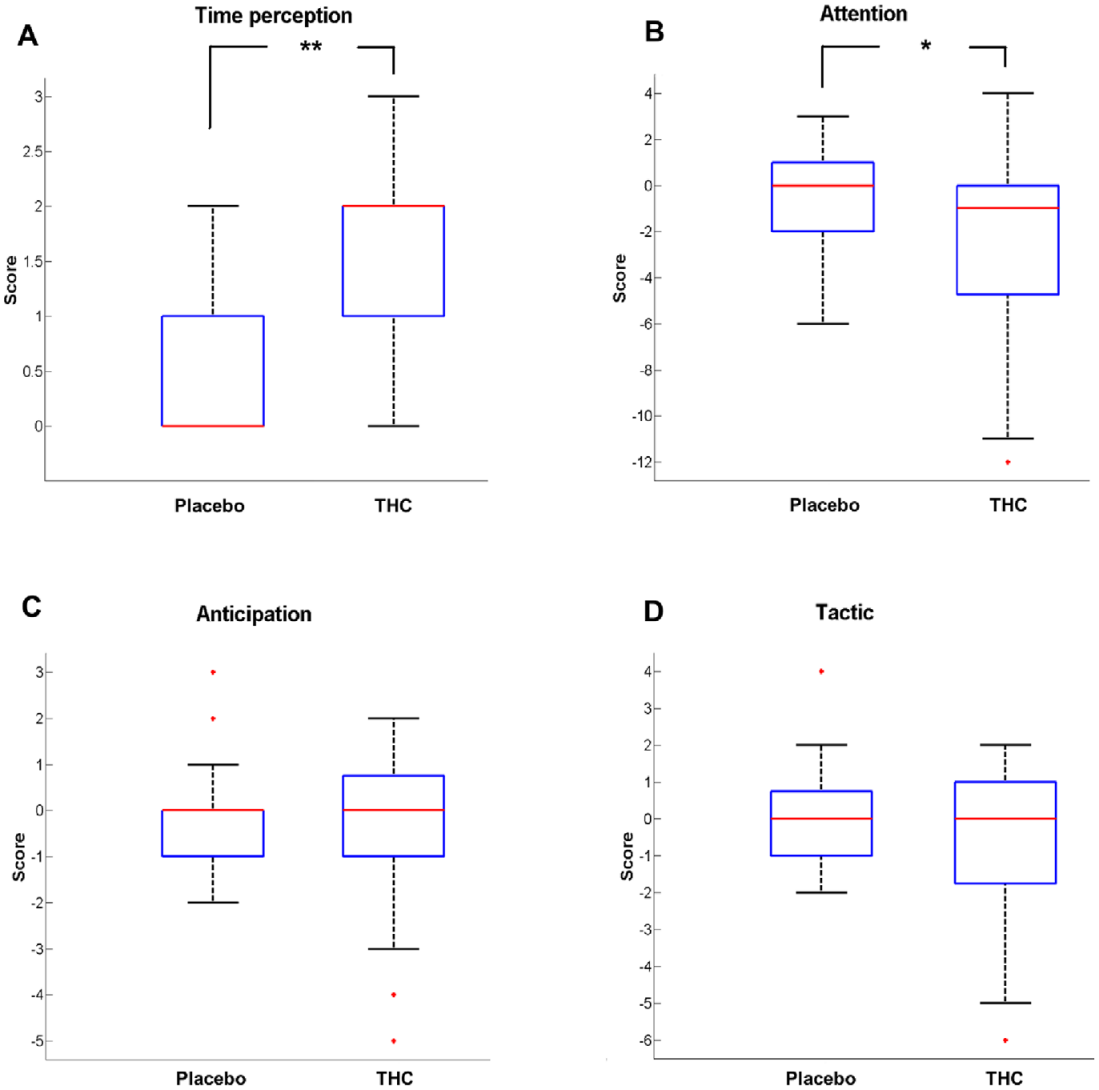
Questionnaire regarding the strategy used to perform the fMRI task. Comparison of the answers given by the volunteers between two experimental sessions (Placebo/THC). The red central mark is the median, the edges of the box are the 25th and 75th percentiles, the whiskers extend to the most extreme datapoints that the algorithm considers not to be outliers (1.5 times the interquartile range), and the outliers are plotted individually with red crosses. Parameters of interest were: alteration in time perception (panel A), attention (panel B), anticipation of the target movement (panel C), and tactic (panel D). Black stars represent the significant difference of the variables of interest between the experimental conditions.

Comparison of differences in time perception assessed before and after cannabis smoking, immediately after each fMRI session, indicated that the judgment of time was significantly altered (p<10^−3^, Wilcoxon signed-rank test). The same comparison revealed that the scores measuring attention or vigilance differences were also significantly different after smoking the cannabis joint than after the placebo (p = 0.04, Wilcoxon signed-rank test) (fig. 4). On the other hand, the same comparison aimed at the detection of changes in the way subjects performed the tracking test did not disclose any significant differences regarding the anticipation of target movements or the tactical approach (fig. 4).

### 3.4. Psychomotor Results, and Correlation with the Subjective Scores

CTT scores measured before and after smoking a placebo or a cannabis joint showed a significant impairment of tracking skills after cannabis smoking (paired T-test, p = 0.01). We observed a slight increase in performance after smoking the placebo (p = 0.04). This increase was ascribed to a learning effect that occurs despite the preliminary training session.

**Figure 5:**
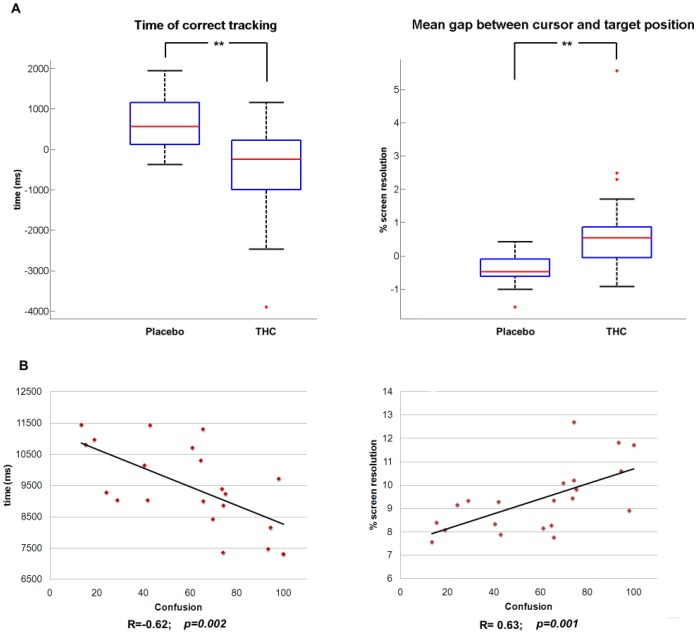
Behavioural results during fMRI session. (A) Comparison of the main behavioural data between two experimental sessions (Placebo/THC). Effects of THC/placebo inhalation were assessed by subtracting the performance during the second post-THC/placebo sessions from the performance during the first control sessions. The red central mark is the median, the edges of the box are the 25th and 75th percentiles, the whiskers extend to the most extreme datapoints that the algorithm considers not to be outliers (1.5 times the interquartile range), and the outliers are plotted individually with red crosses. Black stars represent the significant difference of the variable of interest in the two experimental conditions. (B) Linear correlation between the duration of correct tracking and the feeling of confusion (left panel) and linear correlation between the deviation between target and cursor and the feeling of confusion (right panel). Corresponding Pearson’s correlation coefficients (R) and p-values are displayed at the bottom of each plot.

During the fMRI sessions, the duration of correct tracking in placebo and THC conditions followed a normal distribution. Concerning this behavioural parameter, the paired t-test showed a significant decrease in performance after cannabis smoking compared to the placebo session (p<0.005 corrected). Wilcoxon signed-ranks test revealed statistically significant increase of the mean gap between target and cursor after cannabis inhalation. For this variable, differences between placebo and THC experimental sessions were significant at p<0.005 after correction for multiple comparisons (fig. 5).

Linear regression between the subjective feeling of confusion and the duration of correct tracking revealed a strong negative correlation (R = - 0.62, p = 0.002). In fact, the time during which cursor and target were superposed linearly decreased as the subjective rating of confusion increased. In addition, the gap between target and cursor was positively correlated with the same subjective score (R = 0.63, p = 0.001); the gap linearly increased as the feeling of confusion increased (fig. 5, panel B).

### 3.5. fMRI Results

Group inference was first aimed at assessing brain regions activated while performing the task without any alterations due to either THC or placebo inhalation.

To this end, we analyzed brain regions showing an increase of Blood Oxygen Level Dependent (BOLD) response in the active compared to the passive block during the control session before smoking the placebo (p<0.005, k>40, Doc S2). Clusters in the occipital regions extensively covered primary and higher-order visual areas in both hemispheres. Local maxima were located bilaterally in the middle occipital gyrus, in the inferior occipital gyrus, and in the lingual gyrus. Additional significant clusters were located in the left motor cortex, in the Supplementary Motor Area (SMA), and bilaterally in the cluster extending from the middle frontal to the inferior frontal gyri. Local maxima were located in the central sulcus, in the postcentral gyrus, and in the precentral gyrus. The parietal cortex showed bilateral activation in clusters located in the superior parietal lobule, in the intraparietal sulcus, and in the supramarginal gyrus. Furthermore, we found activation bilaterally in the thalamus, in the insula, and in the left putamen. Doc S2 summarizes each cluster activated, and the corresponding T values.

**Figure 6:**
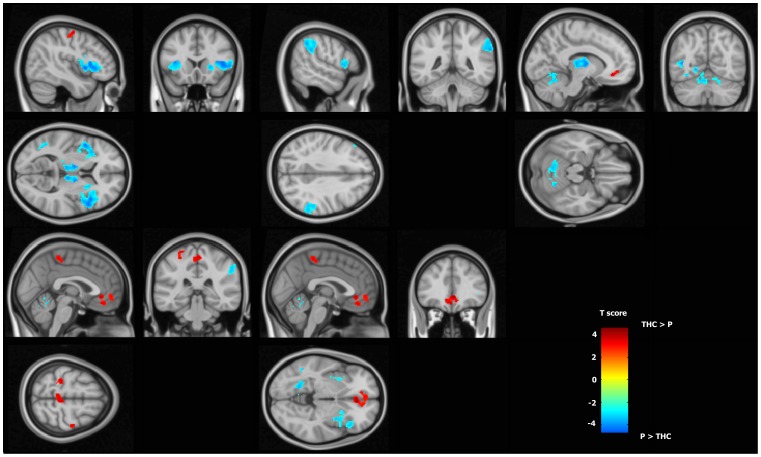
Effect of THC smoking on brain function during the visuo-motor tracking task. When comparing the THC and the Placebo sessions, fMRI BOLD response changes in the Active tracking task vs Passive condition reveal major alteration of brain networks. Hot colour bar represents regions showing an increase in BOLD signal after the cannabis smoking. Cold colour bar represents the opposite contrast. Maps are thresholded at p<0.005 and k>40 and superposed on a standard brain in the MNI (Montreal Neurological Institute) space.

To assess changes in brain activations due to cannabis smoking, we then contrasted the differential maps (Active-Passive) corresponding to placebo and THC conditions (fig. 6). We observed a significant increase in BOLD response compared to placebo smoking in a cluster covering the Anterior Cingulate Cortex and the ventromedial Prefrontal Cortex (vmPFC). The local maximum was located in rostral ACC. The left postcentral gyrus and a cluster covering interhemispherically the SMA showed just a trend (p<0.005 uncorrected). (Table 3).

**Table 3:** Local maxima of significant cluster of activation in the marijuana vs placebo contrast.

Region	Left hemisphere MNI coordinates (mm)			T value	Right hemisphere MNI coordinates (mm)			T value
	x	y	z		x	y	z	
Anterior cingulate cortex	−2	36	−4	4.6				
Postcentral gyrus	−28	−30	56	3.65				
Precentral gyrus/SMA					4	−32	60	3.69

The opposite contrast (Placebo>THC) showed decrease of BOLD signal after cannabis smoking in clusters mainly located in the anterior insula, dorsomedial thalamus, and in the left middle frontal gyrus. Additional clusters were located in the left middle temporal gyrus and in the right superior parietal lobule. The cerebellum showed a trend (p<0.005 uncorrected) (Table 4).

**Table 4:** Local maxima of significant cluster of activation in the placebo vs marijuana contrast.

Region	Left hemisphere MNI coordinates (mm)			T value	Right hemisphere MNI coordinates (mm)			T value
	x	y	z		x	y	z	
Insula	−46	10	6	4.42	46	8	2	4.65
Thalamus	−10	−6	8	4.36	8	−18	6	4.27
Middle frontal gyrus	−46	26	42	4.47				
Middle temporal gyrus	−42	−62	10	4.35				
Superior Parietal lobule					62	−38	46	4.23
Cerebellum	−8	−62	−20	3.43	20	−58	−22	3.66

The addition of alcohol consumption (drinks/week) and the frequency of cannabis use (number of joint/month) as covariates did not influence the activation pattern.

### 3.6. Correlation between BOLD Response and self-estimation of Cannabis Effects

**Figure 7:**
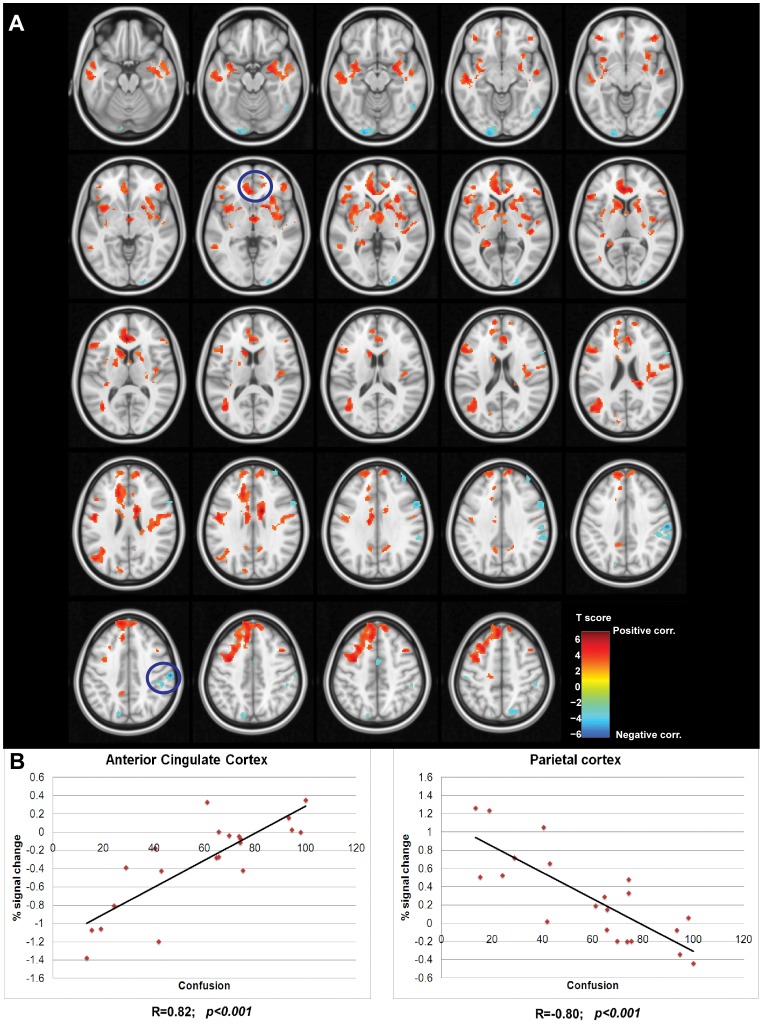
Correlation between BOLD response and the feeling of confusion. (A) Voxel-wise correlation analysis between the BOLD response and the feeling of confusion. Hot colour bar represents regions showing a positive correlation between these two variables, while cold colour bar represents the negative correlation. Maps, thresholded at p<0.005 and k>40 are superposed on a standard brain in the MNI (Montreal Neurological Institute) space and visualized in axial view with slices spaced 3 mm in the z axes. Regions highlighted by the blue circles are the ones plotted in panel B. (B) The left-most plot shows the linear correlation between the mean BOLD response in the cluster located in ACC and the feeling of confusion (p<0.001 corrected). The right-most shows the linear correlation between the BOLD response in the cluster located in the right parietal cortex and the feeling of confusion (p<0.001 corrected). Percent of signal change of BOLD response was averaged across all the voxels belonging to the cluster. Corresponding Pearson’s correlation coefficients (R) are displayed on the bottom of each plot.

Results of the regression between the BOLD response and the feeling of confusion showed the involvement of a network that covers the ACC (rostral and anterior-dorsal), and bilaterally the insula, thalamus, putamen, caudate, nucleus accumbens, DLPFC, superior temporal gyrus, frontal, orbitofrontal cortices, and the left parietal cortex (fig. 7, panel A) that correlates with the subjective feeling of confusion.

The BOLD response and behavioural scores did not show any linear correlation with the blood level of THC during the fMRI assay.

## Discussion

Our study showed that smoking cannabis significantly decreases psychomotor skills and globally alters the activity of the main brain networks involved in cognition even at low concentrations of THC in the blood. Performance and BOLD response didn’t show any correlation with the measured levels of THC but were modulated by the subjective feeling of confusion.

### 4.1. Psychomotor Results – CTT

The CTT detects any impairment present, regardless of its cause, whether from fatigue, alcohol, or cannabis intake. Consequently and in agreement with the guidelines issued by Walsh and coworkers [40], the CTT was used in this controlled study as a reference tracking test. In contrast to the fMRI task which was characterized by a fixed-length of time, and was therefore fully compatible with the block-design of the fMRI experiment, the duration of the CTT depends on the performance of the tested subject. In this experiment, both the critical tracking task (CTT) and the fMRI task yielded the same results: i.e. that on average the tracking performance of the volunteers was significantly and negatively altered after cannabis smoking. This impairment in CTT performance was obvious despite a training effect that tended to conceal its actual full magnitude. Ramaekers et al [5] found that the decrease in CTT performance only occurred in occasional cannabis users and that this detrimental effect extended up to 3–4 hours following cannabis smoking.

### 4.2. Psychomotor Results - fMRI

Consistent with our hypothesis and the validated CTT results, we found that THC exposure significantly decreases task performance as revealed by the psychomotor measurements taken during the active condition of the fMRI. The time period chosen to perform the fMRI task (45 minutes after smoking) is in accordance with the time window of significant impairment after a single dose of THC in occasional cannabis users [4]. The effects of cannabis on brain functions and behaviour extend widely beyond the distribution phase of THC. Performance tests conducted at regular intervals after smoking [5] demonstrated that a single dose of THC impairs tracking performance, divided attention, and inhibitory control in occasional cannabis users. Impairments were maximal during the first hour after smoking and then gradually declined. During the investigated fMRI time-period, THC levels ranged between 2.9 and 23.7 ng/ml (median value 9.4 ng/ml); they were higher than the limits of detection and quantification of our analytical method and close to the technical threshold adopted by authorities for the zero tolerance policy (e.g. 1.5 ng/ml whole blood in Switzerland). These relatively low concentrations, which have detrimental effects on several specific tasks related to driving, are also similar to those found in a previous study [5] and are above the lower limit that was suggested for a significant level of driving impairment (2 to 5 ng THC/ml of serum, i.e. about 1 to 3 ng THC/ml of whole blood) established in a recent paper [41].

### 4.3. fMRI Results

The analysis of the active tracking task during the control session first revealed circuits involved in ocular pursuit, preparation of action, movement, localization, and pointing at a target. We confirmed previous results concerning the existence of polymodal parietal, frontal, and subcortical areas that support cognitive control for selecting, switching among, and attending to salient events in the environment. Such complex activations have already been described in active visuomotor pursuit [42]. Moreover, increased externally driven demand has been associated with increased parietal, pre-motor, and cingular activation [43] and coactivation of the striatum [44].

Recent research suggests that the human brain is organized in different, dissociable intrinsic connectivity networks (ICNs) corresponding to distinct cognitive functions such as vision, audition, sensory-motor, language, episodic memory, executive function, and salience detection [45], [46], [47]. The existence of these independent networks has been revealed in task-free resting state condition [47] as well as during task performance [48]. Changes in BOLD responses are integral to understanding how the activity of three such ICNs – salience, executive-control, and default mode – is altered by cannabis.

Compared to the areas activated during the tracking task in the control condition, cannabis inhalation induced a relative decrease in activation in the anterior insula, the dorsomedial thalamus, the striatum, the right dorsolateral prefrontal cortex (DLPFC), the right superior parietal lobule (RSPL), and the cerebellum. An intuitive explanation would be that this activation decrease is due to an acute impairment of systems important for such a task, i.e. visuo-motor control and motivational striato-frontal dopaminergic systems. This could be supported by the fact that reward pathways, including the dorsal thalamus, insula, and anterior cingulate, are triggered by cannabis cues in addicted people, and this system is certainly modulated by the level of CNB intake [49]. In addicted people, the hypoactivity of the striatum and insula is often associated with hypoactivity of the ACC. This pattern of alteration has been associated with a motivational system where the role of dopamine guides its activity [50]. However, in our study, this relative hypo-activity of the striatum and insula is associated with ACC hyperactivity, and participants are occasional smokers and do not present traits and behaviours peculiar to addiction, as do participants in other cannabis studies. For these reasons, our study doesn’t support a global motivational modification, and orients the interpretation of these alterations toward other mechanisms.

The relative decrease in activation in the anterior insula, dorsomedial thalamus, and striatum is suggestive of a general de-activation of the network implicated in saliency detection. The Salience Network (SN) is a system that detects pertinent environmental changes (regardless of the stimulus modality) in order to guide behaviour. Specific paradigms developed to induce pertinent analysis and motivational salience have been associated with consistent activation of a cortico-subcortical network which includes not only striato-frontal projections, but also the ventral tegmental area (VTA) extending to the bilateral MD thalamus, superior temporal gyrus, posterior insula, and cerebellum [51]. Once the saliencies are identified, the Central Executive Network [45] starts to operate, directing attention to pertinent stimuli. We observed a relative decrease of activation in the right parietal lobule and in the DLPFC that are part of this network [45]. We have shown that both of these networks (SN and CEN) are altered after cannabis smoking; we observed these alterations when participants were performing a demanding visuo-motor task. These alterations might be due to the subjects’ inability to discriminate saliencies, to focus attention, and to behave accordingly.

When looking more closely at the functional role of the discrete regions composing the two networks, the anterior insula (AI) represents a key node involved in switching between brain networks [52]. It has also been shown that the AI has a role in error processing complementary to the ACC since the ACC cannot always differentiate between erroneous and correct response trials [53], [54], [55]. Furthermore, evidence exists that AI plays a crucial role in conscious awareness of errors [17], [56]. The decrease of AI activation under the effect of THC that we observed might then reflect a decrease of subjects’ awareness of their own errors and lower performances.

The cluster located in the RSPL showed a decrease in BOLD response after cannabis smoking compared to placebo and, additionally, a strong correlation with the feeling of confusion (figure 7, panel B). It has been demonstrated that the parietal cortex represents the locus of the neural representation of spatial attention [57], [58]. Furthermore, evidence exists about the involvement of the right parietal cortex in visual search when a manual motor response to a stimulus is required [59]. A recent study also showed greater functional connectivity between prefrontal and occipito-parietal cortex in regular cannabis users as cognitive control demands increased (directing and switching attention, [60]). We explain our BOLD response decrease within the executive network by the lack of recruitment of attention resources.

Cannabis smoking also increased the BOLD signal in the vmPFC and rostral ACC when switching from the passive to the active task. Anatomically, these regions are heavily interconnected with limbic structures and receive a wide range of sensory information from the body and the external environment [61], [62]. It has been shown that a greater activity of the rostral ACC can predict performance errors [63] and that activity with errors during online motor control can reflect a failure in performance optimization [64]. Furthermore, evidence exists about the involvement of the ventro medial prefrontal cortex (vmPFC)/rostral ACC in the judgment of the affective significance of errors and in self-referential mental activity [65]. In fact, the vmPFC is among the brain regions with the highest metabolic rate at rest [66] and as early as 1985 this was attributed to spontaneous self-generated mental activity [67]. Our data might then suggest that cannabis intake favours attention to self-relevant incoming information instead of allocating resources to task-oriented cognitive processing.

An alternative interpretation can be based on the evidence that vmPFC/rostral ACC are parts of the Default Mode Network (DMN). Though further investigation is necessary to fully characterize the psychological and physiological significance of the DMN, it is generally accepted that it represents the baseline activity of spontaneous mental operations that are suspended during goal-oriented behaviour [66], [68]. DMN usually shows a decrease of activity during task performance, and our results show that cannabis seems to impaires DMN inhibition compared to Placebo (Doc S3). Alteration of the functional organization of the DMN in drug addiction has been demonstrated using Resting State fMRI, suggesting diminished cognitive control related to attention and self-monitoring [69], [70]. Greater relative activation (i.e. due to a lack of DMN inhibition) of the vmPFC might also be the cause of increased “self-focused” behaviour [71]. The alteration of time perception, the decrease in the level of attention, and the increase of the subjective feeling of confusion that we observed are in accordance with the hypothesis that subjects are more easily distracted by introspection, with the result of an insufficient allocation of attention resources to task performance.

In this context, the greater activation in clusters located in the pre-SMA and SMA after cannabis smoking must be explained by a compensatory behaviour. These compensatory behaviours require increased motor planning demands and motor regulations, voluntary processes in which the pre-SMA and the SMA play a role [72]. Furthermore, in our study the increase in activation in pre-SMA and SMA is associated with a bilateral decrease of the anterior part of the cerebellum, a region well known to be associated with automatic motor control [73]. After cannabis smoking, subjects need to recruit the SMA more to compensate for the decrease in activation in the cerebellum.

### 4.4. Questionnaires

Among all the questions posed to volunteers, two seemed to best describe the intensity of the effects felt after smoking. The first question concerned the degree of intoxication whereas the second was related to the feeling of confusion. According to Sacco [74], a sense of confusion could result from several factors, such as concentration difficulties possibly triggered by stress and anxiety, and a feeling of depersonalization/derealisation. Furthermore, we found a linear correlation of the feeling of confusion with the BOLD response and the behavioural scores. This has also been demonstrated in chronic users [75].

On the other hand, we failed to find any correlation between the subjective rating of drug effect and the THC levels measured at two time points (right after smoking and levels interpolated during the fMRI exam). A similar observation was made by Toennes [76], and animal studies have shown no correlation of THC levels measured in blood to those measured in the brain [77].

### 4.5. Cannabinoids Elimination Time-profiles

The estimated smoked amount of THC (42 mg) and its concentration in the joint (11% THC, high-grade cannabis) can be considered high if one refers to the numerous controlled administration studies carried out so far with low-grade cannabis joints. Our decision to use high-grade joints relies on the smoking habits of the cannabis users in Switzerland and on the THC concentrations determined in cannabis samples seized by the police. Although the amount of THC that could be inhaled was high, it did not result in blood levels above the usual range of concentrations found in literature. In contrast to serum and plasma, elimination time-profiles of cannabinoids have rarely been determined in whole blood [78]. For comparison, we should consider the cannabinoids distribution ratio between plasma/serum and whole blood [79]. The highest blood concentrations determined in this experiment (median concentration: 87.4 ng/ml (range: 16.8–167.9 ng/ml)) were indeed relatively similar to those typically measured in other experimental controlled studies carried out with occasional users smoking poor and, more rarely, medium-grade cannabis joints [78], [80], [81], where the quality of the joint refers to the cannabis THC content. For instance, Hunault et al [81] indicated that for occasional smokers (between two and nine joints per month), smoking a joint made with a cannabis/tobacco mix containing 49.1 mg THC (cannabis material: 16.4% THC) brought about a maximum THC level of 202.9±112.4 ng/ml of serum (i.e. about 127 ng/ml of whole blood). One hour later, the THC concentration dropped to the 20 ng/ml serum range, matching the THC concentration determined in our study (around 9.4±5.0 ng/ml whole blood (i.e. around 15 ng/ml of plasma)). Other parameters certainly had more influence on blood levels of THC than did the inhaled dose and the concentration of THC in the joints. THC absorption by inhalation is known to be quite variable, with a bioavailability of 2 to 56% through the smoking route depending on depth of inhalation, puff duration, breathhold, and sidestream smoke production [82]. Furthermore, the burning efficiency and vaporization yield of THC contained in pure cannabis joints used in this experiment is lower than that of cannabis joints cut with tobacco [83].

### 4.6. Strengths and Limitations

The main strength of our study relies in the conception of the whole experimental protocol. The timing for biological sampling and psychomotor tests were carefully studied in order to construct reliable kinetic profiles of the major cannabinoids and to put subjects in the most realistic experimental conditions. The time-window chosen for the fMRI experiment is of paramount importance. Since the peak level of THC largely varies across subjects, we decided to perform the fMRI after the rapid distribution phase of THC when cannabinoids concentrations vary less among subjects and decrease slowly (45 minutes after the beginning of the inhalation procedure). This phase was also supposed to correspond to the time-period when drugged drivers are generally apprehended by police. Alteration of brain perfusion due to cannabis has been demonstrated in the literature [20], [22]. We included the passive viewing phase in the design of our fMRI paradigm in order to exclude possible vascular effects due to cannabis intake that are not related to the task. The differential maps (Active-Passive) allowed us to assess brain changes that are only related to the effect of cannabis on the task.

The present study, which was not designed to assess the effects of different doses of THC or the effects on task performances along the whole time-curve of cannabinoids, failed to indicate a statistically significant linear correlation between THC concentrations (at peak level and during the fMRI experiment) and effects on task performances and BOLD signal. A completely different experimental design, including different doses of cannabis and repeated assessments of psychomotor skills along the kinetics of THC, would have needed to have been set up to solve this issue. Also, the visuo-motor pursuit tracking test is not an ecological driving task and this can be a limitation of this study. However, it is a validated task and the correlation with the CTT performances allows our interpretations to be applied to traffic situations.

A limitation of our study is the lack of time-dependent fMRI analysis that can take into account variations in onset time and durations of brain activation between different brain areas or fluctuations of BOLD signal within the blocks. Alternative data-driven methods, with minimal specification of *a priori* constraints, could address this question [84], [85].

Moreover, our approach cannot determine the causal interaction between brain activity and behavioural performance, or the influence of cannabis on their relationship. This point could be addressed only with further investigations, multimodal approaches, or the technique used by Wen and colleagues [86].

In conclusion, we have shown that in occasional smokers cannabis globally altered the activity of the main brain networks involved in cognition despite the low THC concentrations. Subjects might be more attracted by intrapersonal stimuli (“self”) instead of orienting attention to task performance, and this results in an insufficient allocation of task-oriented resources. Effects on BOLD response were associated with the subjective evaluation of the state of confusion. By contrast, we failed to find any quantitative correlation between the THC levels measured in whole blood and either the BOLD signal or the psychomotor performance. These results bolster the “zero tolerance policy” that prohibits the presence of any amount of THC in the blood while driving.

## Supporting Information

Doc S1 **Methods: supplementary material:** Cannabis material, Joint preparation and inhalation procedure, Questionnaires. **Results: supplementary material**: Excluded subjects, Results of the cannabis puffing procedure.
(DOC)

Doc S2 **Control session – Supplementary material.**
(DOC)

Doc S3 **Default mode network – Supplementary material.**
(DOC)
