# Supplementary material for: Weed or Wheel! fMRI, Behavioural, and Toxicological Investigations of How Cannabis Smoking Affects Skills Necessary for Driving
Source: PLoS One. 2013 Jan 2;8(1):e52545. doi: 10.1371/journal.pone.0052545 (PMC3534702; doi:10.1371/journal.pone.0052545)
Supplement: Doc S1 — Methods: supplementary material: Cannabis material, Joint preparation and inhalation procedure, Questionnaires. Results: supplementary material: Excluded subjects, Results of the cannabis puffing procedure. (DOC) [file pone.0052545.s001.doc]

**METHODS - SUPPLEMENTARY MATERIAL**

*Cannabis material*

To be in line with the levels of THC usually measured in marijuana seizures from the Swiss illegal narcotic drugs market (median total THC level: 11.3%, number of seized specimens: 247, second semester 2010, data from the Swiss Society of Forensic Medicine), the joints were rolled with a Dutch cannabis variety containing 11% THC. Permission to import and to use cannabis was granted by the Swiss Office of Public Health (FOPH). Medicinal cannabis (Bedrobinol® variety with a THC-level of around 11% and less than 1% of cannabidiol (CBD)) was obtained from Bedrocan BV medicinal Cannabis, the Netherlands. The Office for Medicinal Cannabis Research (OMC) under the supervision of the Dutch healthcare inspectorate provided export licence and contract. The cannabis variety was supplied in packages of 5 g and was guaranteed to be free of pesticides and heavy metals, and purged of any microorganisms. The THC total concentration (THC-A plus free THC and the CBD total content) was checked by HPLC-DAD after extraction with dichloromethane/methanol 9:1 v/v according to a standard method of analysis . Placebo cannabis (Santhica variety) containing no THC and less than 0.1% CBD was kindly provided by the French National Federation of Hemp Growers (FNPC), Le Mans, France.

*Joint preparation and inhalation procedure*

The cannabis joints were prepared as follows: the mixture of flower-tops and leaves (Bedrobinol® variety or Santhica chemotype) were chopped with a dedicated Zyliss chopper and 0.7g (Bedrobinol variety) or 0.8 g (Santhica chemotype) was added pure into a pre-rolled King Size joint with a 26 mm-long W-folded cardboard filter (Cones King Size 109 mm, 12 Blister Pack, Vandenberg special products, Rotterdam, The Netherlands). The joints containing the active component and the placebo were identical in size and length, and visually indistinguishable.

Participants smoked the joints according to a fixed paced puffing procedure, with 3 seconds getting ready, 2 seconds of inhalation, followed by a breath hold of 5 seconds, and 50 seconds of exhalation and rest. This sequence was repeated until 2/3 of the joint was consumed (up to a line drawn 3 cm above the cardboard filter). The volunteers were allowed to interrupt at any moment the smoking of the joint in case of pronounced unwanted side-effects. The cannabis butt was collected and weighed to estimate the amount of cannabis that had been consumed.

*Biological specimen collection and toxicological analyses*

We collected whole blood samples in order to determine the elimination time-profiles of the major cannabinoids. They were frozen at -80°C in S-monovette® tubes (Sarstedt) containing EDTA/fluoride as preservative agents. Unconjugated cannabinoids ((-)-Δ^9^-Tetrahydrocannabinol (THC), (-)-11-Hydroxy-Δ^9^-THC (11-OH-THC), and (-)-11-nor-Δ^9^-THC-carboxylic acid (THCCOOH)) were measured in whole blood using fast gas chromatography and negative-ion chemical ionization tandem mass spectrometry. Tri-deuterated homologs were used as internal standards. The limits of quantification were 0.5 ng/ml for THC and 11-OH-THC and 2.5 ng/ml for THCCOOH. Cannabinoids were extracted from 0.5 ml whole blood by a rapid liquid-liquid extraction method and then derivatized by using trifluoroacetic anhydride and hexafluoro-2-propanol as fluorinated agents. The method has been fully validated and is routinely used for forensic determination of cannabinoids in blood . Cannabinoids were stable during storage as long as the blood samples were stored frozen and thawed only once.

*Questionnaires*

The participants were asked to say how true the following 10 statements were of them: “I feel high,” “intoxicated,” relaxed,” “I like the effects,” “I feel confused,” “nauseous,” “drowsy,” “dizzy,” “anxious,” and “I feel that my environment has changed.” The overall willingness to drive and the willingness to drive under various fictitious assignments with different emotional load were also assessed with 3 extra statements: “I feel I am able to drive (general),” “I agree to drive a friend to a party (not urgent or necessary),” “I agree to drive a seriously ill child to the hospital (imperative and vital)” . It has been suggested that cannabis smokers might change and adapt their behaviour and driving style to cope with detrimental cannabis side-effects .Our experimental setting didn’t allow any significant modifications in the strategy adopted by volunteers to perform the fMRI task. To confirm this, we used a quantitative self report after each fMRI session in order to detect possible changes in the way subjects performed the tracking tests. With this questionnaire we were able to score the following parameters: level of attention, ability to anticipate target movements, resistance to fatigue, training skills, self-control and impulsiveness, level of understanding, curiosity and liking games, and feeling of altered perception of time, space, and motor skills.

**RESULTS - SUPPLEMENTARY MATERIAL**

*Excluded subjects*

Six subjects were excluded from the research protocol for different reasons (claustrophobia, detection of brain anomalies, or decision to drop out of the study). The smoking protocol was standardized as much as possible to reduce the differences in doses of inhaled THC. Nevertheless, two more subjects were excluded because they did not inhale a sufficient amount of THC to reach the minimal THC threshold concentration of 2.2 ng/ml (2.2 - 30% confidence interval = 1.5 ng/ml) that was interpolated from blood levels measured on both sides of the fMRI time-period. This threshold value was selected taking into account the zero-tolerance policy about drugs and driving and its practical implementation. The Swiss law considers the presence of THC proven when its concentration in the blood reaches or exceeds 1.5 ng/ml. Due to the uncertainty of the measurement ( 30%) the threshold rises to 2.2 ng/ml (Swiss Federal Road Office, 2006).

*Results of the cannabis puffing procedure*

Conjunctival hyperemia was not consistent across subjects. Many of them showed no observable eye reddening after smoking. The temporary increase in the heart rate was more consistent (median value: 81% increase, maximum: 158%, minimum: 30%) while blood pressure remained stable. Such a change in heart rate was not detected after light puffing. Inadequate inhalation had effect neither on the heart rate nor on the THC blood levels, which remained below the 20 ng/ml range.

**References**

1. Hazekamp A, Peltenburg A, Verpoorte R, Giroud C (2005) Chromatographic and spectroscopic data of cannabinoids from Cannabis sativa L. . J Liq Chromatogr Relat Technol 28: 2361-2382.

2. Fournier G, Beherec O, Bertucelli S (2004) Santhica 23 and 27: two varieties of hemp (Cannabis sativa L.) without delta-9-THC. Ann Toxicol Anal 16: 128-132.

3. Thomas A, Widmer C, Hopfgartner G, Staub C (2007) Fast gas chromatography and negative-ion chemical ionization tandem mass spectrometry for forensic analysis of cannabinoids in whole blood. J Pharm Biomed Anal 45: 495-503.

4. Martin NG, Boomsma DI (1989) Willingness to drive when drunk and personality: a twin study. Behavior genetics 19: 97-111.

5. Heath AC, Martin NG (1992) Genetic differences in psychomotor performance decrement after alcohol: a multivariate analysis. Journal of studies on alcohol 53: 262-271.

6. Aitken C, Kerger M, Crofts N (2000) Drivers who use illicit drugs: behaviour and perceived risks. Drugs: education, prevention and policy 7: 39-50.

7. MacDonald S, Mann R, Chipman M, Pakula B, Erickson P, et al. (2008) Driving behavior under the influence of cannabis or cocaine. Traffic Inj Prev 9: 190-194.
