# Supplementary material for: Weed or Wheel! fMRI, Behavioural, and Toxicological Investigations of How Cannabis Smoking Affects Skills Necessary for Driving
Source: PLoS One. 2013 Jan 2;8(1):e52545. doi: 10.1371/journal.pone.0052545 (PMC3534702; doi:10.1371/journal.pone.0052545)
Supplement: Doc S2 — Control session – Supplementary material. (DOC) [file pone.0052545.s002.doc]

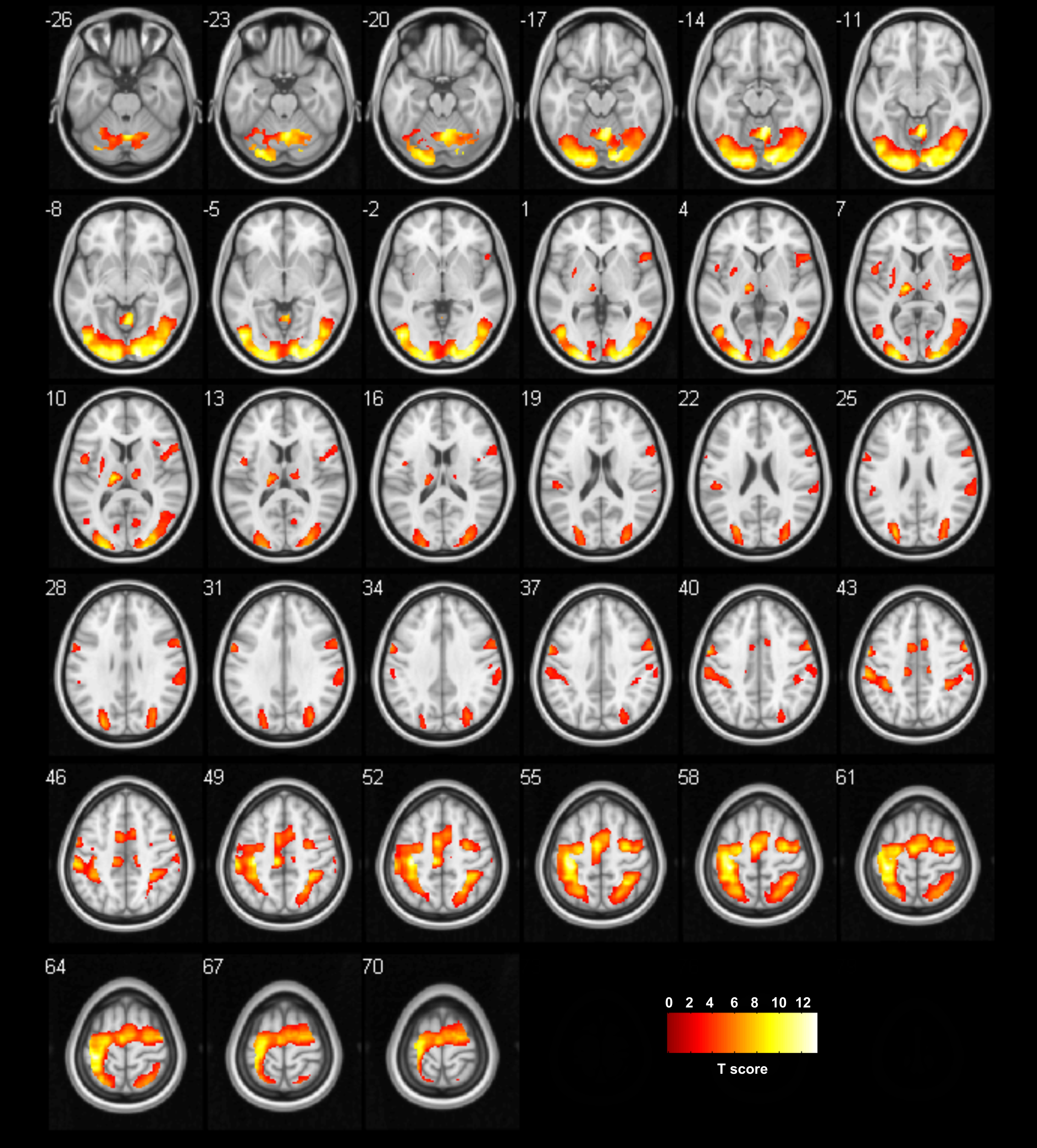
**CONTROL SESSION - SUPPLEMENTARY MATERIAL**

Figure S1. Brain regions recruited while performing the task in the control session. FMRI BOLD response changes in the Active tracking task vs Passive condition in the control session before the smoking of the Placebo joint. Map is thresholded at p < 0.005 and k>40. Maps are superposed on a standard brain in the MNI (Montreal Neurological Institute) space and visualized in axial view with slices spaced 3 mm in the z axes. Hot colour bar represents T scores.

**Table S1. Local maxima of significant cluster of activation during the control session.**

| **Region** | **Left hemisphere MNI coordinates (mm)** | | | **T value** | **Right hemisphere MNI coordinates (mm)** | | | **T value** |
| --- | --- | --- | --- | --- | --- | --- | --- | --- |
|  | **x** | **y** | **z** |  | **x** | **y** | **z** |  |
| Middle Occipital gyrus | -46 | -74 | -2 | 12.49 | 46 | -72 | -4 | 9.44 |
| Inferior Occipital gyrus | -24 | -90 | -12 | 11.88 | 32 | -88 | -4 | 12.73 |
| Lingual gyrus | -14 | -90 | -16 | 11.88 | 14 | -94 | -8 | 13.16 |
|  |  |  |  |  |  |  |  |  |
| Central sulcus | -34 | -32 | 62 | 13.43 |  |  |  |  |
| Postcentral gyrus | -34 | -26 | 52 | 12.79 | 60 | -20 | 46 | 4.19 |
| Precentral gyrus | -26 | -18 | 70 | 9.93 |  |  |  |  |
| Precentral gyrus/SMA | -4 | -8 | 58 | 9.84 |  |  |  |  |
| Middle frontal/Inferior frontal | 56 | 0 | 44 | 8.26 | 56 | 4 | 46 | 7.2 |
|  |  |  |  |  |  |  |  |  |
| Superior Parietal lobule | -24 | -60 | 58 | 13.43 | 30 | -50 | 56 | 10.29 |
| Intraparietal Sulcus | -26 | -82 | 22 | 11.02 | 32 | -76 | 30 | 6.67 |
| Supramarginal gyrus | -48 | -28 | 22 | 4.8 | 62 | -36 | 28 | 4.43 |
|  |  |  |  |  |  |  |  |  |
| Thalamus | -14 | -20 | 8 | 9.16 | 12 | -16 | 10 | 5.45 |
| Insula | -48 | 0 | 10 | 5.17 | 52 | 16 | 2 | 5.1 |
| Putamen | -30 | -2 | 2 | 3.98 |  |  |  |  |
