# Supplementary material for: Weed or Wheel! fMRI, Behavioural, and Toxicological Investigations of How Cannabis Smoking Affects Skills Necessary for Driving
Source: PLoS One. 2013 Jan 2;8(1):e52545. doi: 10.1371/journal.pone.0052545 (PMC3534702; doi:10.1371/journal.pone.0052545)
Supplement: Doc S3 — Default mode network – Supplementary material. (DOC) [file pone.0052545.s003.doc]

**DEFAULT MODE NETWORK - SUPPLEMENTARY MATERIAL**

**
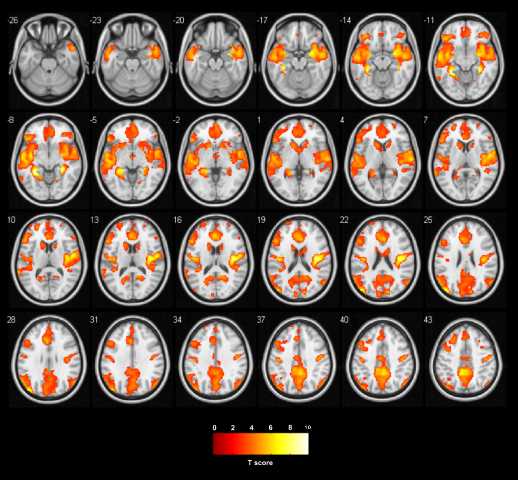
**

Figure S2. Brain regions recruited in the Passive vs Active condition of the fMRI experiment during the control session. Maps are thresholded at p<0.005 and k>40. Maps are superposed on a standard brain in the MNI (Montreal Neurological Institute) space and visualized in axial view with slices spaced 3 mm in the z axes. Hot colour bar represents T scores.

**Supplementary table S2**. Local maxima of significant cluster of activation during the control session in the passive vs active contrast.

| **Region** | **Left hemisphere MNI coordinates (mm)** | | | **T value** | **Right hemisphere MNI coordinates (mm)** | | | **T value** |
| --- | --- | --- | --- | --- | --- | --- | --- | --- |
|  | **x** | **y** | **z** |  | **x** | **y** | **z** |  |
| Inferior frontal /DLPFC | -52 | 28 | 10 |  | 54 | 32 | 10 |  |
| Precentral gyrus | -44 | -16 | 38 | 5.15 | 46 | -12 | 36 | 4.97 |
| Insula | -42 | -14 | 20 | 6.42 | 40 | -16 | 18 | 8.83 |
| Parahippocampal gyrus | -30 | -42 | -8 | 9.87 | 32 | -38 | -10 | 8.3 |
| Middle Occipital gyrus | -44 | -84 | 26 | 7.73 | 56 | -66 | 30 | 5.04 |
| Superior Parietal gyrus/precuneus | -8 | -46 | 44 | 6.98 | 4 | -46 | 42 | 6.59 |
| Amygdala | -26 | -8 | -12 | 6.19 | 30 | -4 | -12 | 6.79 |
| Medial frontal gyrus/MCC | -4 | -14 | 40 |  |  |  |  |  |
| Cuneus | -6 | -76 | 28 | 4.35 |  |  |  |  |
| Superior temporal gyrus | -56 | -12 | 4 | 5.5 | 64 | -16 | 4 | 6.4 |
| Anterior Cingulate Cortex | -4 | 36 | 18 | 6.17 |  |  |  |  |
| Superior medial gyrus | -4 | 30 | 40 | 4.33 |  |  |  |  |
| Mid orbital gyrus |  |  |  |  | 4 | 40 | -10 | 4.12 |
| Middle frontal gyrus | -28 | 26 | 46 | 4.92 | 32 | 24 | 46 | 3.24 |
| Caudate | -10 | 16 | 2 | 4.72 | 14 | 14 | 0 | 4.17 |
